# Supplementary material for: Confidence in naturalistic decision making
Source: Neurosci Conscious. 2026 Jun 19;2026(1):niag024. doi: 10.1093/nc/niag024 (PMC13280941; doi:10.1093/nc/niag024)
Supplement: Supplementary_niag024 [file supplementary_niag024.docx]

**Supplementary Materials**

**Confidence in naturalistic decision making**

Medha Shekhar^1,2^, Axel Cleeremans^2^ & Dobromir Rahnev^1^

^1^ School of Psychology, Georgia Institute of Technology, Atlanta, GA

^2^ Center for Research in Cognition & Neurosciences, ULB Neuroscience Institute, Université libre de Bruxelles, Brussels, Belgium

**Supplementary Figures**

**Supplementary Figure 1. Assessing whether response time (RT) contributes independent information for predicting confidence in our GLM.** To test the necessity of RT as an explanatory variable to predict confidence in our GLM, we fit a “full” model that included the five variables, accuracy and RT and a “reduced” model that only contained the manipulated variables and accuracy as predictors. We compared the full and reduced model by performing a likelihood-ratio test (LRT) which determines whether the added variable in the full GLM has independent explanatory value. We fit the GLM separately for each subject and estimated the average linear regression coefficients, β, for each variable. The plots show the likelihood-ratio (LR) scores between the full and reduced models. For a majority of the subjects (29 out of 57 subjects in Experiment 1 and for 44 out of 58 subjects in Experiment 2), including RT as a predictor significantly improved the model’s ability to predict confidence, suggesting that RTs indeed contribute independently to explaining confidence data. Dots represent individual human participants; the dashed vertical line marks the critical LR value at the 0.05 significance level.

**Supplementary Figure 2. Assessing independent linear relationships between each variable and confidence controlling for accuracy only.** We fit a generalized linear model (GLM) by regressing confidence against each of the five variables while including only accuracy as a predictor in this model to control for its effects. We fit the GLM separately for each subject and estimated the average linear regression coefficients, β, for each variable. (a) For Experiment 1, in which participants were allowed to respond freely, accuracy was the strongest predictor of confidence (Mean β = 0.409, t(56) = 26.573, p < 0.001). In addition, event chronology and group size significantly predicted confidence (Event chronology: Mean β = -0.0189, t(56) = -3.569, p < 0.001; Group size: Mean β = -0.007, t(56) = 4.002, p < 0.001). Specifically, both variables showed a negative relationship with confidence. The coefficients were not significant for any of the other variables – arousal (Mean β = 0.001, t(56) = 0.277, p = 0.783), valence (Mean β = 0.003, t(56) = -0.540, p = 0.591), and context (Mean β = -0.001, t(56) = -0.786, p = 0.435). (b) For Experiment 2, in which participants chose their responses from a fixed set of options, accuracy was the strongest predictor of confidence (Mean β = 0.385, t(57) = 20.327, p < 0.001). In addition, group size also again significantly predicted confidence independent of accuracy (Mean β = -0.010, t(57) = -6.347, p < 0.001). However, we observed no significant effect of event chronology on confidence (Mean β = 0.003, t(57) = 0.564, p = 0.575). Instead, arousal showed a significant negative linear relationship with confidence (Mean β = -0.005, t(57) = -2.886, p = 0.006) and there were no significant effects of valence (Mean β = -0.006, t(57) = -1.647, p = 0.105) or context (Mean β = 0.001, t(57) = 0.557, p = 0.580). Overall, these analyses largely replicate our original findings where the GLM controlled for both task accuracy and RT indicating that our findings are robust. Error bars represent SEM; dots represent individual human participants.

**Supplementary Figure 3. Effect of arousal, valence, event chronology, context and group size on RT.** A) Effect of arousal. (left) Medium levels of arousal facilitated RTs in Experiments 1 but seemed to have no effect for Experiment 2. (right) The quadratic coefficients for RT as a function of arousal were small but significantly positive on average for Experiment 1 but not for Experiment 2 (Experiment 1: mean $a$ = 0.044, t(56) = 2.406, p = 0.019; Experiment 2: mean $a$ = 0.016, t(57) = .434, p = 0.666). B) Effect of valence. (left) Similar to arousal, medium valence ratings also facilitated RTs in Experiment 1 but had no effect on RTs in Experiment 2. (right) The quadratic coefficients for RT as a function of valence were small but significantly positive on average for Experiment 1 but not for Experiment 2 (Experiment 1: mean $a$ = 0.065, t(56) = 2.600, p = 0.012; Experiment 2: mean $a$ = 0.018, t(57) = .683, p = 0.497). C) Effect of event chronology. Similar to accuracy, reversing the sequence of events had no significant effect on RTs in both experiments (Experiment 1: t(56) = -.631, p = .531; Experiment 2: t(57) = -1.262, p = 0.212). D) Effect of contextual information. (left) RTs were facilitated for clips with the lowest or highest amount of context (which were also associated with the highest accuracy and confidence). (right) The quadratic coefficients for both experiments were significantly negative (Experiment 1: mean $a$ = -0.051, t(56) = -5.340, p < .001; Experiment 2: mean $a$ =-.042, t(57) = -3.458, p < .001). E) Effect of group size. (left) In Experiment 1, RTs were facilitated by medium levels of group size compared to extreme levels. On the other hand, in Experiment 2, RTs appeared independent of levels of group size. (right) Quadratic coefficients were significantly positive on average for Experiment 1 but not for Experiment 2 (Experiment 1: mean $a$ = 0.122, t(56) = 4.614, p < .001; Experiment 2: mean $a$ = -.019, t(57) = -.399, p = 0.691). Overall, response times were more significantly affected by the manipulated variables (arousal, valence, context and group size) for Experiment 1 where participants made free choices compared to Experiment 2, where they chose their responses from a fixed set of three choices, suggesting that responses are more likely to be influenced by these variables when choice uncertainty is higher. Note that higher arousal ratings indicate more calmness. Error bars represent SEM; dots represent individual participants.

**Supplementary Figure 4. Assessing the independent contributions of low-level visual features such as RGB, Hue and Contrast in predicting confidence.** We fit generalized linear models (GLMs) by regressing confidence against each of the five variables and accuracy. We fit three separate GLMs where we also included one of three low-level visual features (Hue, RGB values and Contrast) as predictors to control for their effects. The RGB, Hue and contrast values were computed by first extracting these values frame-by-frame and then computing the average over all the frames for each video clip. First, we tested whether each of these low-level features could predict confidence independent of accuracy and the manipulated variables. Therefore, we fit a “full” model that included the low-level visual feature and a “reduced” model that only contained accuracy and the manipulated variables as predictors. We compared the full and reduced model by performing a likelihood-ratio test (LRT) which determines whether the added variable in the full GLM has independent explanatory value. We fit the GLMs separately for each subject and estimated the average linear regression coefficients, β, for each variable. A) Testing the effect of RGB. The plots show the likelihood-ratio (LR) scores between the full and reduced models. For about 90% of the subjects (51 out of 57 subjects in Experiment 1 and for 52 out of 58 subjects in Experiment 2), RGB values failed to add any explanatory value to the model in predicting confidence. B) Testing the effect of Hue. The plots show the LR scores between the full and reduced models. For about 96% of the subjects (54 out of 56 subjects in Experiment 1 and for 56 out of 58 subjects in Experiment 2), accounting for hue was not necessary to explain the effect of variables on confidence. C) Testing the effect of Contrast. The plots show the LR scores between the full and reduced models. For at least 93% of the subjects (53 out of 57 subjects in Experiment 1 and for 54 out of 58 subjects in Experiment 2), contrast did not add any explanatory value to the regression model in predicting confidence. D) It is possible that the lack of explanatory value for low-level visual features in the GLM is due to their intrinsic correlations with other variables in the GLM such as affective features. In other words, changes in one’s valence towards a stimulus may already include changes in their low-level features. For example, bright and colorful stimuli may be perceived as more pleasant. Therefore, we correlated each low-level visual feature with valence scores. Indeed, valence scores were significantly correlated with each low-level visual feature except Hue – Red, Blue, Green and Contrast. Error bars represent 95% confidence intervals; dots represent individual human participants; the dashed vertical line marks the critical LR value at the 0.05 significance level.

**Supplementary Figure 5. Analyzing the stability of each variable’s effect across the first and second halves of Experiment 1**. A) Effect of arousal on confidence and accuracy. As in the main analysis, arousal exerted dissociable effects on confidence and accuracy such that accuracy was highest for extreme (low or high) levels of arousal whereas confidence was highest for medium levels of arousal. Importantly, these effects were stable across the first and second halves of the experiment. The quadratic coefficients for confidence and accuracy were significantly different from each other with the coefficient being positive on average for accuracy and negative for confidence for both halves (Half 1: t(56) = 3.249, p = .002; Half 2: t(56) = 3.950, p = .002). B) Effect of valence. Both accuracy and confidence showed a negative curvature for valence such that they were highest for neutral stimuli. There was no significant difference in the quadratic coefficients for confidence and accuracy for both halves of the experiment, suggesting that its effects on confidence and accuracy were stable (Half 1: t(56) = 0.055, p = .956; Half 2: t(57) = .869, p = .389). C) Effect of event chronology. For both halves of the experiment, reversing the sequence of events had no effect on accuracy (Half 1: t(56) = 1.185, p = .24; Half 2: t(57) = .750, p = .457) but significantly lowered confidence (Half 1: t(56) = 2.883, p = .006; Half 2: t(57) = 2.286, p = .027). Dots represent individual participants and error bars represent the SEM. D) Effect of contextual information. For both halves of the experiment, accuracy and confidence were highest for clips with extreme (low or high) levels of context. The quadratic coefficients were also significantly higher for confidence compared to accuracy for both halves (Half 1: t(56) = -2.787, p = .007; Half 2: t(57) = -2.879, p = .006). E) Effect of group size. Scene complexity exerted dissociable effects on confidence and accuracy such that accuracy tended to be highest for extreme (low or high) levels of group size whereas confidence was highest for low levels of group size. However, the quadratic coefficients for accuracy and confidence were significantly different from each other only for the second half of the experiment (Half 1: t(56) = -.532, p = .597; Half 2: t(57) = 3.518, p < .001). Error bars represent SEM; dots represent individual participants. Overall, we find that the effects of arousal, valence, event chronology and contextual information are stable across time. In contrast, the effect of group size may be dependent on learning or exposure.

**Supplementary Figure 6. Assessing accuracy, confidence, and metacognitive scores for responses scored by Rater 2 in Experiment 1.** a) Participants showed high levels of accuracy (mean = 0.804, SD = 0.089) but average confidence was lower than accuracy (mean = 0.746, SD = 0.100). Mean confidence was lower than accuracy for 46 out of 57 participants, suggesting that most individuals tend to be underconfident. Nevertheless, confidence reliably tracked accuracy with an average Phi score of 0.506 (t = 27.151, p < 0.001). Error bars represent SEM; dots represent individual participants.

b) Average accuracy and average confidence were also significant and highly correlated across individual participants (r = 0.612, p < 0.001). Individual dots represent participants. All the results from responses scored by Rater 1 (in the main text) replicated for the responses scored by Rater 2.

**Supplementary Figure 7. Assessing independent linear relationships between each variable and confidence for responses scored by Rater 2 in Experiment 1.** Accuracy was the strongest predictor of confidence with the largest linear regression coefficient (Mean $\beta$ = 0.396, t(56) = 20.035, *p* < 0.001). Event chronology and scene complexity also both independently showed a negative linear relationship with confidence (Event chronology: Mean $\beta$ = -0.0176, t(56) = -3.440, *p* = 0.001; Scene complexity: Mean $\beta$ = -0.004, t(56) = -2.428, *p* = 0.018). None of the other variables showed any significant linear relationship with confidence. Error bars represent SEM; dots represent individual human participants. All the results from responses scored by Rater 1 (in the main text) replicated for the responses scored by Rater 2.

**Supplementary Figure 8. Assessing non-linear effects of affective states on accuracy and confidence for responses scored by Rater 2 in Experiment 1.** a) (left) Confidence and accuracy as a function of arousal. Accuracy was highest for extreme (low or high) levels of arousal whereas confidence was highest for medium levels of arousal. (right) The quadratic coefficients for confidence and accuracy were significantly different from each other with the coefficient being positive on average for accuracy (mean $a$ = 0.007, t(56) = 2.874, p = 0.006) and negative for confidence (mean $a$ = -0.005, t(56) = -3.347, p = 0.001). The coefficients for accuracy and confidence were also significantly different from each other (t(57) = 4.823, p < 0.001), indicating that arousal robustly exerted an independent non-linear effect on confidence. Note that higher arousal ratings indicate more calmness. b) (left). Confidence and accuracy as a function of valence. Both accuracy and confidence showed a negative curvature for valence such that they were highest for neutral stimuli. (right) The quadratic coefficients were significantly negative on average both for accuracy (mean $a$ = -0.008, t(57) = -3.500, p < 0.001) and confidence (mean $a$ = -0.012, t(57) = -7.755, p < 0.001). There was no significant difference in the quadratic coefficients for confidence and accuracy (t(57) = 1.262, p = 0.212) suggesting that valence exerted similar effects on both variables. Note that higher valence ratings indicate more positively valenced stimuli. All the results from responses scored by Rater 1 (in the main text) replicated for the responses scored by Rater 2. Error bars represent SEM; dots represent individual participants.

**Supplementary Figure 9. Assessing non-linear effects of prior information on accuracy and confidence for responses scored by Rater 2 in Experiment 1.** a) Average confidence and accuracy computed separately for clips that were presented in correct and reverse chronological order. Reversing the sequence of events decreased both accuracy (t(56) = 2.140, p = 0.037) and confidence (t(56) = 4.292, p < 0.001), although the decrease in confidence was larger. Responses scored by both Raters 1 and 2 showed a decrease in confidence for clips in reverse sequence. However, unlike for Rater 2, for the responses scored by Rater 1, there was no significant effect of event chronology on accuracy. b) Confidence and accuracy as a function of context (defined as clip number in the sequence. Later clips are associated with more contextual information). Both accuracy and confidence were highest for clips with extreme (low or high) levels of context. (right) Quadratic coefficients were not significantly different from zero for accuracy (mean $a$ = 0.002, t(57) = 1.245, p = 0.218) but were significantly positive for confidence (mean $a$ = 0.005, t(57) = 5.458, p < 0.001). The quadratic coefficients were significantly higher for confidence compared to accuracy (t(57) = -2.457, p = 0.017), indicating that confidence is independently influenced by contextual information. For the context manipulation, all results from responses scored by Rater 1 (in the main text) replicated for the responses scored by Rater 2. Error bars represent SEM; dots represent individual participants.

**Supplementary Figure 10. Assessing non-linear effects of scene complexity on accuracy and confidence for responses scored by Rater 2 in Experiment 1.** Scene complexity was defined as the number of people in the video clips. (left) Confidence and accuracy as a function of scene complexity. Scene complexity exerts dissociable effects on confidence and accuracy such that accuracy is highest for extreme (low or high) levels of complexity whereas confidence is highest for low levels of complexity. (right) The quadratic coefficients were not significantly greater than zero for accuracy (mean $a$ = 0.003, t(57) = 1.080, p = 0.285) but were significantly negative for confidence (mean $a$ = -0.004, t(57) = -2.284, p = 0.026). The quadratic coefficients were significantly lower for confidence compared to accuracy (t(57) = 2.726, p = 0.009), indicating that scene complexity does exert dissociable effects on accuracy and confidence. All results from the responses scored by Rater 1 replicated for Rater 2, except for the finding of a significant non-linear effect of scene complexity on confidence. For rater 1, the non-linear effect of scene-complexity on confidence was not significant. Error bars represent SEM; dots represent individual participants.
